# Multisectoral action to address noncommunicable diseases: lessons from three country case studies

**DOI:** 10.3389/fpubh.2024.1303786

**Published:** 2024-02-21

**Authors:** Svetlana Akselrod, Téa E. Collins, Daria Berlina, Katia De Pinho Campos, Guy Fones, Diogo de Sousa Neves, Fatima Bashir, Luke N. Allen

**Affiliations:** ^1^World Health Organization, Geneva, Switzerland; ^2^Healthier Systems Ltd., Streatley, United Kingdom; ^3^London School of Hygiene and Tropical Medicine, University of London, London, United Kingdom

**Keywords:** noncommunicable disease (NCD), salt, tobacco, edible oil and fat, multistakeholder and multisector collaboration

## Abstract

**Introduction:**

Multisectoral action is a central component of the global response to the rising prevalence of non-communicable diseases (NCDs). In this paper we aimed to unpack the definition of multisectoral action and provide an overview of the historical context, challenges, and recommendations alongside three country case studies: salt reduction in the UK, tobacco legislation in Nigeria, and regulation of edible oils in Iran.

**Methods:**

We used an iterative review process to select three country case studies from a list of 20 potential cases previously identified by WHO. At our third round of review we unanimously agreed to focus on salt reduction in the UK, tobacco regulation in Nigeria, and edible oil regulation in Iran as these represented rich cases on diverse risk factors from three different world regions that we felt offered important lessons. We conducted literature reviews to identify further data for each case study.

**Results:**

Across the three studies a number of important themes emerged. We found that multisectoral approaches demand the often difficult reconciliation of competing and conflicting values and priorities. Across our three chosen cases, commercial interests and free trade agreements were the most common obstacles to successful multisectoral strategies. We found that early consultative stakeholder engagement and strong political and bureaucratic leadership were necessary for success.

**Discussion:**

The complex multi-rooted nature of NCDs requires a multisectoral approach, but the inevitable conflicts that this entails requires careful navigation.

## Introduction

Noncommunicable diseases (NCDs), including cardiovascular diseases, cancers, chronic respiratory diseases and diabetes are one of the greatest global health and development challenges of our time. These conditions are responsible for an estimated 41 million deaths each year globally, of which 17 million are considered premature mortality, affecting individuals between the ages of 30 and 69 (1). NCDs are also responsible for two thirds of all disability-adjusted life years (DALYs) globally (2). The burden of NCDs disproportionately falls on the most vulnerable population groups. According to WHO, 77% of all NCD deaths and 85% of premature deaths occur in low-and middle-income countries (LMICs) (1).

Despite the growing attention to NCDs at the global level (3) and the inclusion of NCD-related targets in the Sustainable Development Goals (SDGs)—notably target 3.4 which calls for a one-third reduction of premature mortality from NCDs and promotion of mental health and well-being by 2030 (4, 5)—current evidence suggests that most countries are not on track to achieve these targets (6).

A key challenge to NCD prevention and control is the fact that the main drivers and risk factors—including poverty, air pollution, physical inactivity, and the marketing and sale of tobacco, alcohol, and processed foods—all lie beyond the sphere of control of government health departments or national health systems (7). The responsibility for addressing these underlying determinants sits across multiple sectors and government departments, such as education, employment, transportation, trade, finance, environment, agriculture, and manufacturing. The rising global burden of NCDs is forcing policymakers to engage with and work across these non-health sectors.

The call for multisectoral action for health is not new. The 1978 Declaration of Alma Ata highlighted the role of determinants that lie outside the control of the health sector and established intersectoral action as crucial for achieving health for all. The 1986 Ottawa Charter for Health Promotion called for “healthy public policies” at every level of government (9). To address structural determinants of health and to narrow widening global inequities, the WHO Commission on the Social Determinants of Health recommended that “policies and programs must embrace all the key sectors of society not just the health sector” (7). This includes engaging with individuals with lived experience as key stakeholders, as well as private companies whose operations influence NCD risk factors and health outcomes.

The Sustainable Development Goals (SDGs) present an inherently multisectoral approach to sustainable development. Achieving SDG Target 3.4 on NCDs and mental health by 2030 requires multisectoral and multistakeholder engagement, greater policy coherence, coordination and collaboration across all government departments and society at large (10–12). More explicitly, SDG Goal 17.6 calls for global multistakeholder partnerships to support sustainable development. To include the private sector, SDG 17.17 encourages the promotion of effective public, public-private and civil society partnerships, and SDG 17.14 highlights the importance of improving policy coherence across all government sectors (5, 13).

The WHO guiding framework on Multisectoral Action for the prevention and control of NCDs (14) identifies the elements necessary for the success of multisectoral initiatives and is based on four pillars:

1. Governance and accountability: The degree and commitment of government leadership to foster multisectoral and multistakeholder engagement to tackle NCDs and create a collaborative culture within and across different sectors and actors.
2. Leadership: Understanding of how governance structures can be leveraged and/or created to foster an enabling environment and mandate for multisectoral action.
3. Clear and purposeful ways of working: The tools and processes used to implement and operationalize multisectoral and multistakeholder action.
4. Resources and capabilities: The resource needs (both human and financial) to support multisectoral and multistakeholder initiatives.

## Aims

In this paper, we aimed to describe common challenges to scaling up multisectoral and multistakeholder action for NCD prevention and control and provide several case studies analyzed according to the WHO guiding framework on multistakeholder action.

## Methods

Our study team sought to identify a small number of county case studies, from diverse settings, that highlighted different experiences of multistakeholder collaboration and multisectoral action. We use the term “multisectoral action” interchangeably with “intersectoral action,” both referring to any form of collaboration within and across government sectors. The term “multistakeholder action” implies any form of synergistic engagement between government, the private sector, civil society (including non-governmental organizations and academia), and international development partners with intersecting interests, to further a collective aim (8). We discussed a long-list of 20 potential case studies that had been produced by the internal WHO team as part of a prior exercise. Our criteria for inclusion in the study were based around the richness of the information and evidence available for each case study, the number of different sources available, the potential for learning lessons, and the degree to which the case study concerned a generalizable NCD problem, e.g., control of a major risk factor rather than action to increase the availability of a particular medicine. After two rounds of review we collaboratively ruled out 15 of the potential case studies, almost all of which had relatively little information or only one source. Based on wordcount constraints we decided to aim for three case studies, balancing the wordcount available for each case against the desire to represent multiple world regions and NCD risk factors. At our third round of review we unanimously agreed to focus on salt reduction in the UK, tobacco regulation in Nigeria, and edible oil regulation in Iran.

We conducted literature reviews to identify relevant evidence for each case study. This included web searches; searching the websites of the stakeholders involved in each initiative; and searching the references of relevant papers. We also had access to data that had been submitted on each case study to WHO during a call for evidence on multisectoral action on NCDs. Our search strategy included the country name AND synonyms for the relevant risk factor with additional searches using relevant dates, names of actors, and the name of the policy initiative. We screened titles and where these were relevant or unclear we screened abstracts/the first webpage. Information was included from all articles and webpages that presented information on governance and accountability, leadership, ways of working, resources and capabilities or descriptions of the events.

We used the framework analysis approach described by Spencer and Richie (21). This approach is well suited for policy relevant qualitative research where the objectives of the project are set in advance (22). We deductively coded the data using four domains based on those of the WHO guiding framework on multisectoral action: governance and accountability, leadership, clear ways of working, and resources and capabilities. We used the same concepts as the WHO framework for each domain, but expanded our scope to consider the degree and commitment of leadership from any relevant stakeholder, rather than just focusing on the level of government leadership. As we progressed and new gaps or potential themes emerged we sought out additional relevant data using web searches and discussion with subject experts linked to WHO. We could not always find evidence that perfectly aligned with each of the four framework domains for each of the three case studies. Where that was the case, we flagged the evidence gap. We presented our findings narratively and drew on the wider literature from other sectors to develop a series of recommendations on how to successfully use multisectoral action to address NCDs.

## Findings

### Case study 1: multistakeholder collaboration to reduce salt intake in the United Kingdom

Limiting dietary salt intake is considered one of the most cost-effective interventions to tackle cardiovascular diseases (23). WHO Member States have committed to reducing the mean population salt intake by a relative 30% and achieving 25% relative reductions in blood pressure by 2025 (3). The UK has implemented one of the most successful and comprehensive salt reduction programs involving the public, private, and civil society sectors.

Starting in 2003, the UK established a voluntary salt reduction collaboration with the food industry, which was promoted by the non-governmental organization *Consensus Action on Salt and Health* (CASH, now known as *Action on Salt*) and the Food Standards Agency (24). The most instrumental component of the program was the collaboration between the Department of Health, the quasi-governmental Food Standards Agency, private food industry partners, and CASH. Using the WHO multisectoral action framework, the elements that facilitated this successful outcome can be summarized as such:

- *Governance and accountability*: In 1996, an action group of 22 experts on salt and blood pressure was formed (CASH) in response to the government rejecting a series of salt reduction recommendations. The government’s rejection was felt to reflect stiff industry opposition. CASH pursued significant advocacy efforts toward both the Chief Medical Examiner of the UK and successfully persuaded the Food Standards Agency to shoulder the responsibility for salt reduction as part of its broader work on nutrition (24) in collaboration with the UK Department of Health. As such, the accountability mechanism was initiated from the bottom-up by an NGO.
- *Leadership*: The government did not play a leadership role. Instead, an academic and civil society coalition took the initiative. Their work identifying champions from both the private and public actors was key to the success of this multisectoral collaboration. The CASH group lobbied for salt reduction initiatives with private food industries such as Asda, Marks and Spencer, and Sainsbury’s as well as identifying a key player within the government to take the initiative further, namely the Public Health Minister. CASH was able to find champions within the private sector who were willing to join their cause and advocate for other corporate groups.
- *Clear and purposeful ways of working*: The group’s strategy included setting voluntary salt targets for different categories of food with clear time frames and progressively ambitious targets for the industry to achieve. They worked to gain consensus around targets that balanced feasibility with maximum ambition. CASH also engaged with food manufacturers to encourage reformulation toward lower salt levels, implemented consumer education strategies, and introduced regular gold-standard monitoring through 24-h urinary sodium measurements in random population samples (24). From 2004, the Food Standards Agency with input from CASH and other stakeholders developed a model to examine the impact of reducing the average salt composition of food products on the population’s salt consumption. In 2006, they published the first set of salt targets to be achieved by 2010 (over 4 years), aiming for a 10–20% reduction in mean salt intake every 1–2 years. The decision to aim for incremental reductions was based on the premise that smaller reductions are not detectable to consumers and therefore minimize the threat to sales. The food industry welcomed the fact that the targets applied to all producers, creating a “level-playing field” where all companies had to comply equally to reduce the salt content in any particular food. The Food Standards Agency and partners worked very closely with the food industry to reach this consensus. Initial work on the reformulation program was complemented by a consumer awareness campaign to raise awareness of the risks associated with salt intake (25). There were no legal ramifications for missing the targets, however, the high profile of the collaboration and marked media interest meant that many manufacturers were concerned about being “named and shamed” for not complying (24).
- *Resources and capabilities*: CASH benefitted from the input of a wide range of respected subject experts but did not have any dedicated funds. Instead, the early phases of this drive for reform depended entirely on research staff supported by other grants. In later years significant funding was provided by the UK government and other charitable organizations including the British Heart Foundation.

To date, four sets of voluntary salt reduction targets have been published (in 2006, 2009, 2011, and 2014). In 2014 average salt intake in the UK was estimated to be approximately 8 g/day with only a third of the population estimated to be meeting the 6 g/day consumption target. In 2014, 76 targets were published for the food industry to meet by the end of 2017. Both average and maximum targets were set. In 2018, Public Health England, published an in-depth analysis to assess whether the food and drink targets set in 2014 were met. The results showed that just over half (52%) of all the average salt reduction targets set were met by 2017. Retailers made more progress than manufacturers, meeting 73% of the targets compared with manufacturers meeting approximately 37%. Overall (for both retailers and manufacturers combined), where maximum targets were set, 81% of products had salt content levels at or below their set targets (retailers 86%, manufacturers 72%). While falling short of the desired impact, this evidence shows that since 2004 there has been clear and significant progress in reducing population salt intake.

More recently, Public Health England published the 2024 salt reduction targets for all sectors; retailers, manufacturers, restaurants, takeaways and deliveries. The new targets include revised average and maximum targets, as well as new targets for foods not previously covered. The revisions of targets have been a consultative process. In February 2020, stakeholders were invited to input and feedback on the revised targets. A prominently expressed concern from all industry stakeholders was that some of the targets may not be feasible due to commercial and/or technical challenges. Considering the reductions already achieved, some argued that there was limited room for further reduction (26).

Even though the ultimate WHO goal of 5 g/day salt intake has not been reached, overall, the impact of the UK salt reduction program is still significant considering the short time frame. A recent systematic review showed that the UK achieved a 7% reduction in salt intake from the period 2005–2019 from 8.1 to 7.5 g/day (27), and 2.7-mm Hg fall in average population systolic blood pressure, and a significant reduction in mortality from stroke and ischemic heart disease that is largely attributable to the fall in salt intake (28, 29). While acknowledging the fact that safe salt levels have still not been achieved, the UK salt reduction program can be considered an example of a successful multistakeholder approach to an important NCD risk factor, driven by civil society, and bringing together stakeholders from public and private sectors to work collectively toward a shared goal. Over time, responsibility for the reform shifted from CASH to the UK government, which also assumed full responsibility for adequately resourcing the policy. There is a gap in the evidence around exactly how and why this transfer of ownership happened.

### Case study 2: passing the 2015 national tobacco control act in Nigeria

Despite Nigeria’s ratification of the Framework Convention on Tobacco Control in 2005, it took a full decade before a comprehensive tobacco bill was passed. A National Tobacco Bill was introduced in 2009, aiming to “provide regulation for the control of production, manufacture, sale, advertising, promotion and sponsorship of tobacco or tobacco products” (30). The bill was sponsored and supported by local politicians but strongly opposed by British American Tobacco, Nigeria (BATN) and other industry advocates who actively sought to halt the bill from passing. The bill was held up for 4 years and ultimately failed In 2013 due to further pressure from the tobacco industry.

However, just 2 years later the government passed an Executive Bill later known as the “National Tobacco Control Act 2015” (NTCA) (31). The bill is a comprehensive, FCTC-complaint legal instrument that encompasses all the Tobacco “best buy” interventions in addition to other measures that relate to the reduction of the demand and supply for tobacco. Multisectoral action was central to the formulation and passing of the bill; involving 16 sectors that ranged from government ministries and agencies to NGOs and civil society including religious groups, but excluding the private sector and the tobacco industry (30). This is irrespective of the fact that the Standard Organization of Nigeria, which regulates the production of manufactured goods and products, called for the involvement of the tobacco industry, and subsequently involved the tobacco industry in the development of the 2014 Standard for Tobacco and Tobacco Products-Specifications for Cigarettes (30, 32). This was justified based on the expectation that policy is expected to guide the manufacturing practices of the tobacco industry (30).

Using the multisectoral action framework, the elements that facilitated this successful outcome can be summarized as such:

- *Leadership*: The bill received high-level leadership and the endorsement of President Goodluck Jonathan at the time, despite the formidable backlash and opposition of BATN. This political endorsement may have reflected a bid to garner support from the bill’s stakeholders ahead of a re-election campaign, nonetheless, this support was instrumental in the successful passage of the bill (33).
- *Governance and accountability*: The NTCA as a legal instrument provided the authorizing environment and mandated for multisectoral action. The bill addressed fundamental elements of tobacco control through multisectoral and multistakeholder action via establishing a tobacco control committee and the tobacco control unit; and including measures such as tobacco control funding; prohibition of smoking in public places and tobacco advertising, prohibition of tobacco product sales to minors; regulation of the contents of tobacco products; tobacco products packaging and labelling; enforcements and roles of a responsible organization; public awareness; price and tax measures among a few others (33).
- *Clear and purposeful ways of working*: Among the most critical and influential factors that aided the passage of the Act was the pressure from civil society and NGOs. Furthermore, the understanding among members of the tobacco control committee is that the Federal Ministry of Health alone could not implement the tobacco control actions proposed in the bill without the involvement and contribution of other relevant ministries and organizations. There was a general perception that the involvement of multiple relevant sectors was required to produce effective tobacco legislation that was in line with their interests (30). The collective exchange of ideas from involved stakeholders increased the sense of joint ownership and laid a foundation for collaboration and potential sustainability (30).
- *Resources and capabilities*: The president’s office lent the requisite technical capability needed to draft and pass the Bill. There were no direct costs involved.

While the NTCA is a successful example of how multistakeholder and multisectoral action can lead to significant efforts in furthering tobacco control, there has been Implementation proved to be challenging (33). For example, while the NTCA provides that the textual warnings on cigarette packages will be reviewed and updated at least every 2 years, this has not yet happened and the warning messages prescribed under the 1990 law are still in use. Also, while BATN complies with the NTCA provision of textual warning, the print is barely legible and covers <50% of the total surface area of the packet in conflict with the policy provision (33). Furthermore, the tax rate on tobacco products stands at 28% and is considerably lower than the WHO recommendation of above 70% (34).

### Case study 3: regulation of edible oils in Iran

To address NCDs in Iran, close collaboration between the public sector, private health associations and the food industry has led to the successful adoption of a number of national and international dietary targets. The Food and Drug Administration is responsible for national food safety and, as a central member of the national NCD committee, the Administration works closely with industry players in food and nutrition sectors to improve the effectiveness of food policies and strategies (35). To minimize the adverse effects of unhealthy diets on the prevalence of NCDs, a multistakeholder consultation was conducted that included regulators and academic researchers. This led to a series of recommendations around some staple foods including oil products. Recognizing the significant carcinogenic effects of palm oil derivatives that arise from the refining process, the Iranian Food and Drug Administration worked with the oil industry to substitute palm oil with other vegetable-based oils. The main strategy was setting a mandatory maximum permitted limit of palm oil content in the most commonly used household frying oils and industrial frying oils (35, 36). The following factors were key to the successful implementation of the mandatory limit:

- Governance and accountability: The Iranian Food and Drug Administration, as the central body responsible for food safety played a critical role in the success of the multisectoral initiative by bringing together stakeholders from all relevant fields and sectors. The collaboration in setting national targets for food components was imperative in setting the adjusted national targets and in turn, for compliance by the food industry. The presence of a principal agency mandated with food security not only allowed for the promotion of a multisectoral initiative but created a form of public accountability for all stakeholders to abide by.
- Leadership: Nesting this multisectoral initiative under a primary government agency provided momentum for the initiative and created a culture of collaboration among involved stakeholders. This clear and centralized form of leadership facilitated the creation of professional networks across the different sectors.
- Clear ways of working: The tools employed were based on rigorous academic principles whereby international guidelines and recommendations on food intake were studied by relevant stakeholders including academics and regulatory experts (35). Using an evidence-based approach to identifying targets will create a challenge for stakeholders particularly the industry sector, to refute the needed changes to food content. The availability of evidence strengthened the case for editing national targets.
- Resources and capabilities: The initiative was funded and led by the Food and Drug Administration, which had the resources to bring together the relevant stakeholders, and the technical capability to convene the relevant evidence and develop an appropriate policy and regulatory framework.

As a result of the initiative, the maximum permitted levels of saturated and trans fatty acids in food were modified in the national regulations. Consequently, the addition of palm oil to foods was restricted in national food industries to safe levels. Today, Iranian oil industries use various oil fractions to produce food products which generally consist of about 70% common vegetable oils, up to 25% palm oil, and a maximum of 5% of fully hydrogenated oils. The recent formulations contain higher unsaturation rates and fewer trans and saturated fatty acids (35).

## Discussion

We found a number of common factors that drove progress across the three case studies. The first is the importance of having clear and committed leadership from one stakeholder group. While the WHO Framework views leadership through a governmental prism, the UK example shows that a reform agenda can be driven by civil society. At some point government has to “buy in” in order to pass the relevant policy, but the minister of health does not necessarily have to be the project’s main champion. Having said that, the Nigerian case study shows what can happen when a head of state takes a personal stake in policy reform: Goodluck Jonathan singlehandedly revived a policy that was thought to be “dead in the water.” Even so, Nigeria’s case study demonstrates the necessity of working with a coalition of stakeholders to develop and pass a well-designed policy. The fact that the policy has been poorly implemented provides another important lesson, and chimes with the UK salt situation where success against the odds has yet to translate to safe levels of salt consumption. The process of building momentum and coalitions should not stop at the point where a policy is passed; instead the focus should be on achieving the desired health outcome—the policy is a means rather than an end in itself.

All of the case studies emphasize the importance of recruiting and winning over allies in a range of sectors. The CASH group strategically identified the key actors and then engaged with these groups. Stakeholder mapping can be used in this way to identify those with power to bring about the desired change (e.g., government lawmakers) as well as other actors who hold influence over these people and their likely position (e.g., for or against reformulation). An understanding of the key actors is an important basis for developing public health strategy.

For salt reduction strategies, evidence suggests that comprehensive strategies that involve “upstream” population-wide policymaking such as reformulation and product labelling (as opposed to individual-level or downstream policymaking) can achieve the greatest public health gains. The UK salt reduction initiative combined multi-component strategies of both upstream and downstream approaches awareness campaigns, co-agreed target settings for salt reduction, and voluntary reformulation from the food industry (37). An instrumental factor that contributed to the UK salt initiative was the incremental and progressive reduction in salt targets coupled with an effective monitoring strategy as this approach minimized the impact on industry (24). This was also the case for Tunisia, where a pilot reduction initiative in collaboration with local bread-making businesses was successful in reducing the average salt content in the bread-making process by approximately 30% during the first 3 months of the pilot (an average of 10% reduction per month) and was able to maintain reduction for the subsequent 33 months totaling an average of 35% reduction in salt consumption after the three-year pilot intervention. Salt was gradually reduced throughout this period by 35% to a level that was undetected by consumers and did not affect consumer appreciation of the bread (38, 39). This “win-win” outcome is encouraging and similar progressive, incremental approaches can be adopted for large-scale salt reduction programs at national levels.

Reformulation is an effective measure often employed by government agencies and refers to altering the composition of processed foods, e.g., reducing salt and fat composition. From a policy formulation perspective, this can be a “voluntary” or “mandatory” reformulation. While both approaches are typically government-led, the main difference is the latter involves the use of legal channels to enforce compliance. Many public health critics argue that voluntary measures fall short in terms of implementation efficiency and stronger “mandatory” measures are necessary to ensure maximum impact (40). Portugal introduced a salt reduction strategy that was modeled on the UK multistakeholder collaboration; however, the industry representatives successfully diluted the salt reduction targets and extended the implementation deadlines. Modeling has shown that watering down the targets has caused over 250 additional premature deaths from cardiovascular disease each year (41). Similar failures have been seen with trans fat voluntary regulation in Canada and in New York, where voluntary efforts to reduce trans fat use in restaurants were introduced but showed no impact after 1 year of implementation, while a legal ban led to near-elimination (42). Similarly, voluntary trans fat reduction interventions in six European countries (Slovenia, Croatia, Serbia, Bosnia, and Herzegovina) found high concentrations of industrially-produced trans fats still present in many food items (43).

The UK’s alcohol policy approach provides another example of where voluntary co-regulation has not been successful in aligning health policy needs with industry preferences. The UK’s “Public Health Responsibility Deal” intended to bring together stakeholders from a variety of sectors including the alcohol industry, public health NGOs, and government health policymakers, to jointly work toward public health goals through collaboration and voluntary action—replicating the “success” of the salt collaboration (44, 45). While the intention was to “work collaboratively” to agree on policy options for the alcohol industry, fierce divisions between the public health and alcohol industry aim have undermined any meaningful progress.

South Africa provides an example of where mandatory measures have been used to improve the food supply, particularly in setting salt limits. South Africa’s mandatory salt limits were signed into law in 2013 by the Minister of Health, requiring production to adhere to an initial sodium limit by 2016 and a second lower limit by 2019 (40, 46). While the limits had regulatory backing, they had been developed through a consultative multistakeholder process. This multisectoral approach also benefitted from consistent political leadership, guidance from international NCD policy experts, and the use of robust scientific evidence (47). It is too early to determine the success as the policy is still under implementation however recent data suggests that approximately two-thirds of the food items covered by the law have already met the limits of sodium reduction (40).

Mixed results have been seen for other food components. The Iranian case highlighted above was an example of a successful collaboration between multiple stakeholders to lower the food content of palm oil by industry producers, Similar collaborations have been tried in Zambia, Rwanda, Uganda and Namibia for implementing sugar reduction policies, but in all strong industry, resistance has undermined effective action (48–51).

The momentum created by the SDGs has contributed to the proliferation of multisectoral and multistakeholder (i.e., public, private and third-sector actor) engagement initiatives around NCDs and other global health priorities. These build on foundations laid by the 66th World Health Assembly, as it endorsed the WHO Global Action Plan for the Prevention and Control of NCDs 2013–2020, calling for a multisectoral approach to tackling NCDs that utilize a multistakeholder perspective. In 2019, the WHO Global Action Plan was extended to 2030 by the Seventy-second World Health Assembly to align with the 2030 Development Agenda (57).

While multisectoral and multistakeholder actions are increasingly being promoted to maximize health benefits and achieve health equity, both face challenges, particularly in resource-constrained settings. One major barrier is related to the human workforce capacities in the public health sector, specifically the lack of competencies and leadership skills needed to work across sectors and with diverse stakeholders, and abilities to adequately assess and manage risks to ensure public health efforts are not tainted by competing interests (47).

From a governance perspective, challenges to effective multisectoral collaborations include the absence of convening and coordinating mechanisms and adequate administrative and legal structures; conflicting interests and agendas across sectors, including competition for limited public budgets; lack of appropriate accountability and transparency mechanisms; weak data collection and monitoring processes; miscommunication and mistrust across sectors; and incoherent policies across government department (17, 58, 59). The subject of “health ownership” is also of extreme relevance to multisectoral action, as many non-health sectors view health policy as “owned” by the Ministry of Health, creating much hesitancy toward engagement. Furthermore, non-health sectors may view these activities as imposing upon a “health agenda,” a phenomenon known as “health imperialism” that creates further reluctance among non-health sector partners (15).

There are often competing priorities and conflicts in potential outcomes between and within sectors, departments, organizations, and individual stakeholders, for example, the need to balance desired health gains against economic growth. For governments, a particularly difficult terrain to navigate lies beyond the public sector, especially those of corporations that profit from selling unhealthy commodities (16).

Challenges also arise when governments and health organizations have to engage with sectors and partners that have imperfectly aligned mandates. In many settings, this is further compounded by weak governance structures and the overwhelming economic influence of the private sector and industries on national economies, as well as financial influence over individual politicians (17). The scientific community, NGOs, and other civil society organizations may play a significant role in shaping the regulation environment that can counter this strong commercial influence.

The private sector exerts influence through various tactics including marketing, lobbying, corporate social responsibility policies and a predominant role in supply chains, domestically and globally (18). Moreover, globalization and international trade agreements can limit the scope of what national governments can do in terms of industry regulation (19). Negotiations for trade in which the health dimensions are not carefully observed and considered can create hurdles for national regulatory capacities and jurisdiction for setting laws and regulations aligning with national NCD reduction targets. Strong leadership and clear policy guidance on how to manage engagement with the private sector are necessary to streamline and maintain effective multisectoral and multistakeholder initiatives (20).

The global trade liberalization agenda has generally led to increased consumption of tobacco, alcohol, and processed foods that are typically higher in salt, fat and sugar contents (52). International trade agreements and international organizations regulating trade (e.g., the WTO) have significant impacts on the extent to which national governments can impose health-related policies, despite public health carve-outs. On the one hand, health agencies have a direct duty to ensure the health of their population and prevent and control disease, while trade agencies and authorities are primarily concerned with trade regulation, domestic and international. These often very conflicting mandates and interests make it difficult to use a multisectoral approach to address diet-related causal factors for NCDs. For example, when the Thai Food and Drug Administration proposed a law intended to reduce children’s consumption of unhealthy snack foods, by labelling food items, this was challenged by WTO state members which delayed the proposed regulation and subsequently led to an industry-preferred “Guideline Daily Amount” labelling option becoming mandated instead (53). A recent quasi-experimental analysis has shown that participation in US and European Union (EU) free trade agreements is associated with a significant reduction in the implementation of several WHO-recommended NCD policies that target tobacco, alcohol, and unhealthy foods (54). The European Commission has also previously prevented countries from introducing public health policies on anti-competition grounds (55).

The absence of an international agreement on alcohol or unhealthy foods cannot fully explain the inaction on multisectoral approaches for NCDs. National governments possess the power to formulate, prioritize and operationalize their national health priorities regardless of any international agreements and/or frameworks. National Strategic Plans (NSPs) are usually the blueprint for countries when developing budgeted strategic priority areas of action for the health sector. Multisectoral approaches should be an essential element of every NSP when developing NCD action plans. Embedding multisectoral approaches, goals and action plans into the NSP is an expression of political commitment and finances toward multisectoral action. For example, increasing the price of alcohol (via taxation) is the most effective strategy to prevent negative health effects from alcohol consumption but this requires significant policy coherence, e.g., a tax policy needs support from ministries of finance, trade, and consumer protection agencies (56), and codifying these policies in the NSP would to a large extent signal buy-in from these ministries and provides a route for establishing accountability measures. This is always balanced with the degree of influence of the alcohol industry in a given economy and its ability to sway policymaking. Health policymakers must be better equipped to understand and interact with this political dynamic to ensure effective multisectoral action strategies that involve industries can be effectively designed and implemented. Embedding this in a national strategy or action plan may be a mechanism to operationalize multisectoral approaches for NCDs in a sustainable and enforceable manner.

### Recommendations for governments and public health leaders

Government and public health leaders must be equipped with the skills and competencies required to play an active role in multisectoral collaborations. Strong political leadership is essential to ensure alignment and coherence across all government sectors (58). Indeed, attracting the interest of non-State actors is less challenging when a given public health problem is consistently and credibly presented by high-level political leaders as carrying significant costs to society in terms of both health outcomes and economic costs. We would argue that this scenario was evident during the COVID-19 pandemic and the earlier HIV/AIDS outbreak, as well as in recent campaigns to address emerging issues such as anti-microbial resistance. Government leaders and public health officials should keep in mind that multisectoral collaborations can start with voluntary compliance but will be more effective and sustainable in the long term if voluntary compliance is complemented by binding agreements and regulations. Upstream interventions that are more comprehensive and population-targeted are more efficient in comparison to downstream or individual-level interventions that rely on individual preferences and/or perceptions.

Political commitment alone, while admirable, will not suffice. National governments must reflect this commitment into actionable budgeted national programs that explicitly promote multisectoral action approaches as a key strategy to tackle NCDs. A critical component to operationalizing a multisectoral NCD action plan is the early engagement of relevant stakeholders, while safeguarding public health from undue influence. Resources must also be dedicated to multisectoral and multistakeholder efforts, for example establishing working groups or forum where all partners are involved and consulted. Early engagement increases buy-in from partners and improves the prospects of commitment and adherence to agreed plans. Furthermore, national plans must include robust monitoring and evaluation mechanisms that allow for continuous monitoring of implementation to identify and address gaps and maximize impact.

In practice, when aiming to engage with non-State actors for NCD prevention and control, governments should start by conducting a stakeholder mapping exercise to determine how and to what extent various entities align with and add value to the NCD-related public health goals. It is also important to note the nuances when engaging with cross-sectoral government agencies or non-State actors; the mode of engagement, mandates, risks, and accountability frameworks that may differ significantly across actors. Government leaders must choose between different engagement modalities, depending on the purpose of engagement, government’s capacity, the actors involved, the level of engagement desired, and estimated risks. Stakeholders should jointly identify specific metrics to measure success based on established public health goals, ideally monitored by a third party to ensure objectivity. Further, partners should agree upon and spell out the engagement details and develop mechanisms to ensure adherence to commitments and results and enhance all parties’ accountability and transparency.

Global momentum through global declarations and country commitments to global NCD targets are prerequisites for more productive engagement. For national governments, this may mean revising trade agreements and creating mandatory regulations to ensure NCD goals are met. There is an entry point for the public health community to improve trade and health policymaking by actively engaging with related trade negotiations and exploring how this can be leveraged to promote NCD prevention.

Finally, any multisectoral initiative for NCD prevention and control must be carefully tailored to specific country contexts. Ensuring cultural appropriateness is of extreme importance, as public health relies first and foremost on the trust and buy-in of the population it is trying to serve. Any breach of public trust can seriously hinder the intended intervention (60).

## Conclusion

The complex multi-rooted nature of NCDs requires a multisectoral approach with multistakeholder engagement as a core component. However, multistakeholder engagement necessitates working alongside actors and sectors with conflicting interests and ambitions. Furthermore, governance structures and trade agreements often limit the scope of what governments and regulatory authorities can impose. In other cases, governments bow to industry pressure and fail to translate their commitment to the NCD reduction agenda into actionable programs.

Evidence suggests that a successful multisectoral approach to NCD reduction should be aimed at upstream population-wide interventions with less reliance on downstream individual-level initiatives and/or a combination of both. Furthermore, trade agreements must be revisited to remove obstacles that impede NCD reduction strategies. A consultative approach that seeks to engage stakeholders in the early stages of policy development has shown to be more productive down the line producing stronger commitments to shared targets. Mandatory regulations are much more effective than voluntary measures. Finally, strong political commitment and leadership are key and central to any multisectoral NCD reduction program.

## Data availability statement

The original contributions presented in the study are included in the article/supplementary material, further inquiries can be directed to the corresponding author.

## Author contributions

SA: Writing – review & editing, Conceptualization. TC: Conceptualization, Writing – review & editing, Funding acquisition, Project administration, Supervision, Writing – original draft. DB: Conceptualization, Project administration, Writing – review & editing. KP: Writing – review & editing, Writing – original draft. GF: Writing – review & editing. DS: Writing – review & editing. FB: Writing – review & editing. LA: Writing – review & editing, Formal analysis, Methodology, Project administration, Supervision.
